# Preferential and Increased Uptake of Hydroxyl-Terminated PAMAM Dendrimers by Activated Microglia in Rabbit Brain Mixed Glial Culture

**DOI:** 10.3390/molecules23051025

**Published:** 2018-04-27

**Authors:** Yossef Alnasser, Siva P. Kambhampati, Elizabeth Nance, Labchan Rajbhandari, Shiva Shrestha, Arun Venkatesan, Rangaramanujam M. Kannan, Sujatha Kannan

**Affiliations:** 1 Department of Anesthesiology and Critical Care Medicine, Johns Hopkins University School of Medicine, Baltimore, MD 21205, USA; yossefalnasser@gmail.com (Y.A.); eanance@gmail.com (E.N.); 2 Center for Nanomedicine, Wilmer Eye Institute, Department of Ophthalmology, Johns Hopkins, University School of Medicine, Baltimore, MD 21205, USA; skpramodh@gmail.com; 3 Department of Neurology, Johns Hopkins University School of Medicine, Baltimore, MD 21205, USA; labchanr@gmail.com (L.R.); shivashrestha09@gmail.com (S.S.); avenkat2@jhmi.edu (A.V.)

**Keywords:** PAMAM hydroxyl dendrimers, activated microglia, mixed primary glial cultures, intracellular trafficking, enhanced cellular uptake, neuroinflammation

## Abstract

Polyamidoamine (PAMAM) dendrimers are multifunctional nanoparticles with tunable physicochemical features, making them promising candidates for targeted drug delivery in the central nervous system (CNS). Systemically administered dendrimers have been shown to localize in activated glial cells, which mediate neuroinflammation in the CNS. These dendrimers delivered drugs specifically to activated microglia, producing significant neurological improvements in multiple brain injury models, including in a neonatal rabbit model of cerebral palsy. To gain further insight into the mechanism of dendrimer cell uptake, we utilized an in vitro model of primary glial cells isolated from newborn rabbits to assess the differences in hydroxyl-terminated generation 4 PAMAM dendrimer (D4-OH) uptake by activated and non-activated glial cells. We used fluorescently-labelled D4-OH (D-Cy5) as a tool for investigating the mechanism of dendrimer uptake. D4-OH PAMAM dendrimer uptake was determined by fluorescence quantification using confocal microscopy and flow cytometry. Our results indicate that although microglial cells in the mixed cell population demonstrate early uptake of dendrimers in this in vitro system, activated microglia take up more dendrimer compared to resting microglia. Astrocytes showed delayed and limited uptake. We also illustrated the differences in mechanism of uptake between resting and activated microglia using different pathway inhibitors. Both resting and activated microglia primarily employed endocytotic pathways, which are enhanced in activated microglial cells. Additionally, we demonstrated that hydroxyl terminated dendrimers are taken up by primary microglia using other mechanisms including pinocytosis, caveolae, and aquaporin channels for dendrimer uptake.

## 1. Introduction

Dendrimers are unique nanodevices with great potential as drug delivery vehicles and diagnostic tools [1,2]. Their tree-like architecture offers many advantageous characteristics such as monodispersity, biocompatibility, multivalency, and customizable chemical properties [3,4]. In particular, polyamidoamine (PAMAM) dendrimers have been successfully used as drug, gene, and peptide delivery vehicles, as well as contrast agents [5,6,7].

Delivery of drugs across central nervous system (CNS) barriers such as the blood-brain barrier (BBB) and the blood-retinal barrier (BRB), to specifically target cells in the brain, is a major challenge for nanoparticles [8,9,10,11,12,13]. Huang et al. illustrated the ability of modified PAMAM dendrimers to cross the intact BBB [14]. Our group has reported that PAMAM dendrimers, particularly generation 4 hydroxyl-terminated dendrimers (D4-OH), upon systemic administration can cross the BBB/BRB and selectively target and co-localize in activated microglia/macrophages in the brain and retina [11,15,16,17,18] without the need for targeting moieties. Furthermore, systemic dendrimer-drug therapies have shown significantly enhanced efficacy in attenuating neuroinflammation than free drugs in multiple small and large animal models [11,17,18,19,20]. This enhanced cellular uptake and efficacy can be attributed to (1) the size and surface functionality of D4-OH, (2) the ability of D4-OH to cross impaired CNS barriers, and (3) the combined effects of increased phagocytic activity of activated microglia/macrophages and the intrinsic targeting ability of dendrimers.

We have previously shown that the intracellular uptake and trafficking of dendrimers with different surface charges follow different cellular pathways [21]. Generation 4 PAMAM dendrimers (neutral, anionic, and cationic) majorly employ non-specific, clathrein-independent fluid phase endocytosis. However, it is known that different cells employ different endocytosis mechanisms, which may be further modified in the presence of an inflammatory stimulus. Additionally, in vitro studies have reported that dendrimer-drug treatment resulted in enhanced anti-inflammatory activity in LPS-activated microglial cells, compared to free drugs [22,23,24,25]. This can be attributed to increased cellular uptake of dendrimer-drug conjugates in activated microglial cells compared to free drugs that may typically utilize a receptor-mediated process. In multiple preclinical brain injury models, there was a significant increase in the activated microglia and astrocyte uptake of D4-OH PAMAM compared to healthy controls [26,27]. However, the mechanisms by which this uptake occurs and the differences between ‘activated’ and ‘quiescent’ glial cells remain unexplored. Although it has been reported that the intracellular internalization of PAMAM dendrimers occurs via endocytosis, further investigation to determine the mechanisms of increased uptake by activated glial cells is necessary [28,29]. Recent studies have shown that besides the three key factors (surface charge, molecular weight, and generation), PAMAM dendrimer internalization may also be dependent on the targeted cell type [20,30].

In this study, we hypothesized that glial cells utilize more than one route of cell entry for uptake of hydroxyl-terminated PAMAM dendrimers and that the uptake mechanisms may vary depending on the activation status of the cell. We also hypothesized that cell internalization is time-dependent and varies between the different types of glial cells. We labelled D4-OH dendrimers with near IR fluorescent dye cyanine 5 (Cy5) and studied their uptake by primary glial cells using quantitative and qualitative measures. Various pharmacological inhibitors for different cell entry pathways were adopted to clarify the mode of glial cell internalization of the dendrimer. Understanding the mechanism of glial cell uptake will provide information necessary to further optimize dendrimer-based drug delivery vehicles for targeting specific glial cells in the presence of disease pathologies.

## 2. Materials and Methods

DMEM low glucose media (Corning Cellgro, Manassas, VA, USA); HI-FBS, Penicillin/Streptomycin (antibiotics) and DAPI (Invitrogen, Carlsbad, CA, USA); glass-bottom culture dishes (MatTek, Ashland, MA, USA), poly-L-lysine hydrobromide, genestine, sucrose, acetazolamide, and amilioride (Sigma-Aldrich, St. Louis, MO, USA); anti-Glial Fibrillary Acidic Protein Fluor 488 (GFAP) (eBioscience, San Diego, CA, USA); and Tomato Lectin (Vectorlabs, Burlingame, CA, USA) were purchased. The synthesis of Cy5-labelled D4-OH dendrimers has been established and reported previously [17]. Lipopolyscaaharides from *E. coli* 0127:B8 (lot#081M4071V) was purchased from Sigma-Aldrich.

### 2.1. Primary Glial Cell Culture and Cell Treatment

All procedures used in this study were approved by the Johns Hopkins University Animal Care and Use Committee and followed according to approved animal protocols. The cerebral cortices from PND2 New Zealand white rabbits were excised, meninges were removed carefully, and cortices were suspended in 5 mL of 0.05% trypsin for 15 min. The trypsin reaction was neutralized using Dulbecco’s Modified Eagle’s Medium (DMEM) low glucose medium (Corning Cellgro, Manassas, VA, USA) supplemented with 20% heat inactivated fetal bovine serum (HI-FBS) (Invitrogen Corp., Carlsbad, CA, USA) and 2% antibiotics (penicillin/streptomycin) (Invitrogen Corp., Carlsbad, CA, USA). The cortices were minced and triturated into small pieces to separate the cells using sterile cell culture pipettes. The cell suspension was filtered through a 0.2 μm sterile cell strainer (BD Biosciences, San Jose, CA, USA) to remove debris and fibrous layers. The filtered cell suspension was centrifuged at 1000 rpm for 5 min at 4 °C, and the pellet was resuspended in DMEM medium containing 4.5 g/L glucose and 1.4 mM L-glutamine (Corning Cellgro, Manassas, VA, USA) with 10% FBS and 1% antibiotics. The cells were plated into glass-bottom culture dishes or 12-well plates coated with poly-L-lysine hydrobromide (Sigma Aldrich, St Louis, MO, USA) and incubated at 37 °C and 5% CO_2_ atmosphere. Medium was changed every two days, and cells were allowed to reach 90% confluence (day 9–13). Subsequently, planned wells and dishes were treated with LPS in culture medium at 100 ng/mL for glial cell activation. Following overnight incubation with LPS, cells were treated with D-Cy5 at 20 µg/mL with or without cell uptake inhibitors to evaluate the mode of cellular entry. 

To study the mechanism of primary glial cell uptake, cells were initially pretreated with inhibitors to block specific cell uptake pathways, followed by D-Cy5 treatment. The inhibitors used were (1) genistein at a concentration of 100 nM to block caveolae-mediated endocytosis, (2) sucrose at 450 nM to impede fluid phase endocytosis, (3) amiloride at 10 μM to prevent macropinocytosis, and (4) acetazolamide at 100 nM to obstruct aquaporin channels. The inhibitors were dissolved in DMEM medium and incubated with primary glial cells for one hour prior to treatment with D-Cy5.

### 2.2. Cell Cytotoxicity Assay

The effects of the inhibitor treatment on cell viability were evaluated by MTT assay (Invitrogen, Grand Island, NY, USA). Metabolically active cells reduce the yellow tetrazolium MTT in part by the action of dehydrogenase enzymes, to generate reducing equivalents such as NADH and NADPH. The resulting intracellular purple formazan is solubilized and quantified by spectrophotometry to determine the fraction of viable cells. Briefly, primary glial cells were seeded at 10^4^ cells/well in 96 well-plates incubated for 24 h and then treated with the cell inhibitors and LPS, followed by D4-OH dendrimer treatment. The MTT assay was done as previously described by our group and as per manufacturer instructions [22]. Absorbance was read at 540 nm using a micro-plate reader (SynergyMix, BioTek, Winooski, VT, USA) and percent viability compared to untreated controls was calculated.

### 2.3. Cell Imaging

Cells in glass-bottom culture dishes were used at day 8–12 of primary glial cell culture. After treatments, cells were washed with dPBS twice and fixed using 4% paraformaldehyde for 15 min. Tomato Lectin (1:500) (Victorlabs, Burlingame, CA, USA) was co-incubated with anti-GFAP (1:500) (eBioseceince, San Diego, CA, USA) overnight at 4 °C to stain microglia and astrocytes, respectively. The cells were washed twice with dPBS for 5 min, stained with 4′,6-diamidino-2-phenylindole (DAPI) (1:1000) (Invitrogen, Grand Island, NY, USA) for 15 min, and imaged under an LSM 710 confocal microscope (Carl Zeiss, Hertfordshire, UK) for identification of the dendrimers in microglia and astrocytes. A 633 nm laser was used to image dendrimer (D-Cy5) localization, while Tomato lectin and GFAP imaged by 594 and 488 lasers, respectively. The laser intensity levels were kept identical and constant for all the images for appropriate comparison. Images were acquired at 20× magnification and were processed using Zen software (Carl Zeiss) at identical settings. For semi-quantitative analysis, cell signals intensities were analyzed utilizing ImageJ software (NIH, Bethesda, MD, USA).

### 2.4. Flow Cytometry

At various time points (30 min through 24 h), dendrimer-treated cells along with untreated controls were isolated using 0.05% Trypsin. Cells were spun down to pellet out at 1500 rpm in an Eppendorf benchtop centrifuge for 5 min and washed once with FACS buffer (1× PBS with 10% FBS). Each condition contained 1 × 10^5^ cells, which were stained for 30 min on ice with mouse anti-rabbit Fluorescein *Lycopersicon Esculentum* (Tomato) Lectin antibody (1:500, Victorlabs, Burlingame, CA, USA) in 100 μL of FACS buffer to label microglia. For labeling astrocytes, Anti-Glial Fibrillary Acidic Protein Fluor 488 (GFAP) (1:500, eBioscience, San Diego, CA, USA) was used at similar conditions. Unstained glial cells were used to obtain gating data according to their light scattering characteristics. After incubation, both non-labeled and labeled Lectin and GFAP cells were washed once by adding 1000 μL of FACS buffer and centrifuged at 1500 rpm for 5 min before re-suspending in 250 μL of FACS buffer. The total cell population to FL-3 channel (Cy3, Lectin) to find out the microglial population and to assess the percentage of microglia taking up dendrimer was evaluated by gating the lectin +ve cells and analyzing the D-Cy5 fluorescence in the FL-4 channel. A total of 30,000 events was collected for each condition using a BD Accuri C6 Flow Cytometer (BD Biosciences, San Diego, CA, USA). Analysis of fluorescence was performed using CFlow software.

### 2.5. Statistical Analysis

Data were analyzed using analysis of variance (ANOVA) followed by student’s *t*-test using Prism Graphpad. A *p* value less than or equal to 0.05 was considered statically significant. The graphs were constructed using KelidaGraph version 4.1.1 (Synergy, PA, USA). The values are represented as means ± standard error of mean (SEM).

## 3. Results

### 3.1. Selective Uptake of Dendrimers by Microglia in Primary Mixed Glial Cultures

**Figure 1:**
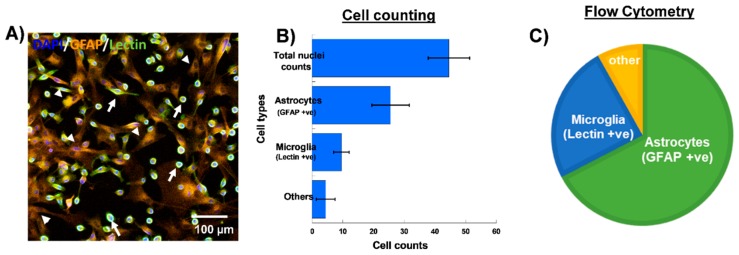
**Characterization of mixed glial cell culture from neonatal rabbit brains.** (**A**) Confocal microscopy image of mixed glial culture showing microglia/macrophages (**Green** stained with lectin, white arrows), astrocytes (**Orange**, stained with GFAP, white arrow heads) and the cell nuclei stained with DAPI (**Blue**). (**B**) Evaluation of different cell population using cell counting from confocal microscopy images. (**C**) Evaluation of percentages of different cell populations present in the mixed primary glial culture using flow cytometry. Scale bar 100 µm.

We used mixed glial cultures derived from neonatal rabbit cortices to investigate the cellular uptake kinetics of the D4-OH. Before beginning the dendrimer uptake experiments, we analyzed the purity of glial cultures using flow cytometry and cell counts using confocal microscopy images. We used GFAP for labelling astrocytes and tomato lectin for labelling microglia/macrophages. Flow cytometry analysis revealed that ~60–70% of the cells in the mixed glial culture were astrocytes (GFAP +ve both in flow and imaging), ~20–25% were microglia/macrophages (lectin +ve both in flow and imaging), and ~8–10% were found to be other cell types (+ve DAPI and –ve GFAP and lectin). The numbers were similar upon cell counting from confocal microscopy images (Figure 1A–C). The other cells may be oligodendrocytes and fibroblasts, since we expect neuronal cells will not survive in our culture conditions, as they require neuronal specific growth supplements and medium.

**Figure 2:**
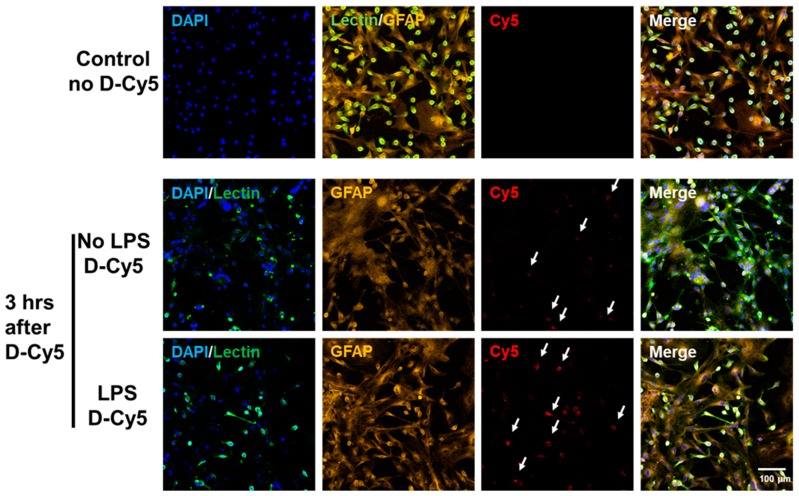
**Preferential and selective cellular uptake of G4-OH dendrimers (D-Cy5) activated microglia/macrophages.** Cy5-labeled D4-OH PAMAM dendrimers (**D-Cy5**) were used to evaluate the selective uptake of dendrimers by microglial cells. Microglial cells stained with lectin (Green), Astrocytes were stained using GFAP (Orange), and nuclei were stained using DAPI (Blue). The activated microglial cells (LPS) group demonstrated a higher number of microglial +VE for dendrimers (**D-Cy5**, white arrows) compared to resting microglia (No LPS). Interestingly, the signal intensity of D-Cy5 is higher in activated microglial cells compared to the D-Cy5 intensity in resting microglial cells, suggesting higher amount of dendrimer uptake. The signal intensities for all channels are kept constant for all conditions. DAPI/Lectin channel is used to better demonstrate the microglia/macrophage population in mixed glia culture taken-up dendrimers, as shown in Cy5 channel. Scale bar 100 µm.

**Figure 3:**
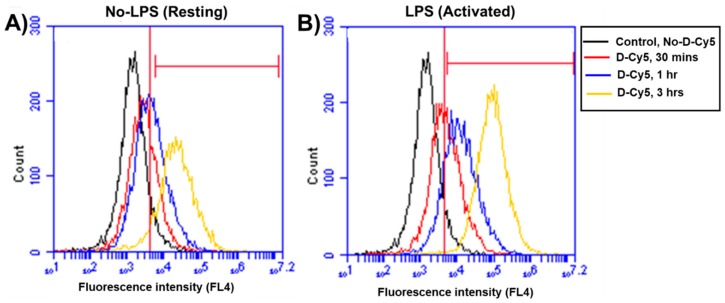
**Flow cytometry analysis of time-dependent differential uptake of dendrimer (D-Cy5) by lectin stained microglia/macrophages population in mixed glial culture.** (**A**,**B**) Histograms of lectin +Ve cells (Microglia/macrophages) showing minimal D-Cy5 uptake (Red) at 30 min in both resting and activated conditions compared to controls (Black). Shift in histograms of lectin +Ve cells (Microglia/macrophages) show increased dendrimer uptake at 3 h. A larger percentage of the activated microglia/macrophage population (~85%) shows D-Cy5 uptake, whereas only ~60% of cells show D-Cy5 uptake in the group without LPS treatment.

**Figure 4:**
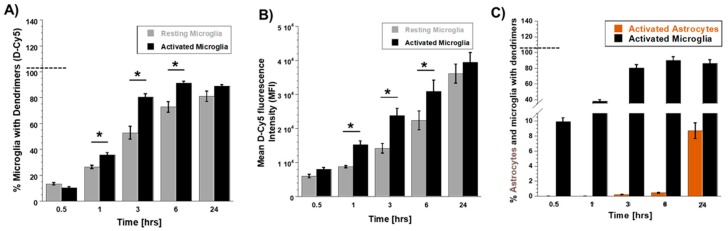
**Evaluation of differential dendrimer (D-Cy5) uptake by resting and activated microglial cells in mixed glial culture.** (**A**) Flow cytometry analysis of time-dependent differential uptake of dendrimers by resting (non-LPS treated) and activated (LPS treated) microglia/macrophage populations. (**B**) Mean intensity fluorescence (MIF) measurements of FL4 channel showing differential amount of dendrimer uptake or intracellular D-Cy5 in resting and activated microglial cells. (**C**) Delayed and limited dendrimer uptake by astrocyte population (~8.5%) 24 h post dendrimer treatment. All the values are represented as mean ± SEM, *n* = 4, * *p* < 0.01, student *t*-test.

**Figure 5:**
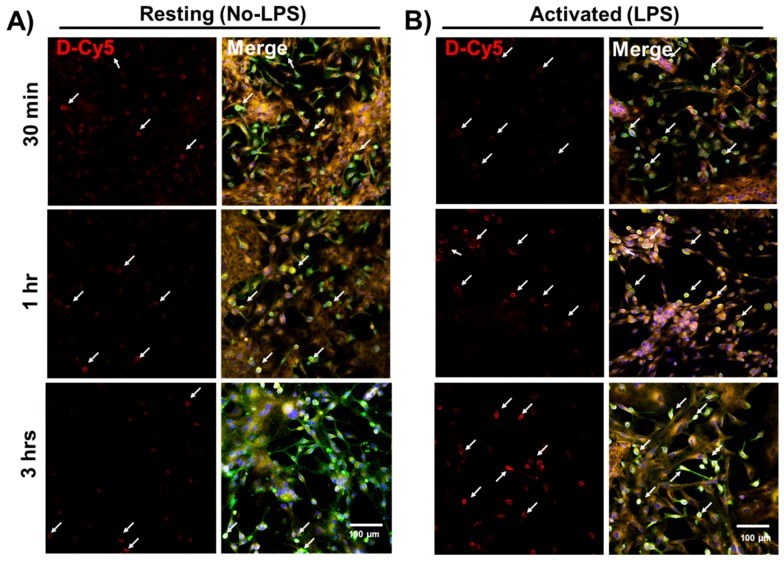
**Time-dependent (30 min–3 h) and differential dendrimer uptake between resting and activated microglial cells in mixed glial cultures from neonatal rabbit brain.** The mixed glial cultures were activated using LPS (100 ng/mL) and treated with 20 µg/mL of D-Cy5. At different time points (0.5, 1, 3, 6, and 24 h) post-D-Cy5 treatment, the cells were fixed and stained with lectin for microglia (Green), GFAP for astrocytes (Orange), and DAPI for nucleus (Blue). (**A**) Resting (no-LPS) microglia/macrophages show less dendrimer uptake compared to (**B**) activated (LPS) microglia/macrophages at all time points (white arrows). Scale bar 100 µm.

We used varying D-Cy5 concentrations (1, 5, 10, 20, 40, and 100 μg/mL) to investigate dendrimer uptake. We found out that a concentration of 20 μg/mL of D-Cy5 was optimal, as increasing the concentration beyond that caused high fluorescence signals and difficulty in evaluating the time-dependent uptake. Dendrimer (D-Cy5) was selectively taken up by microglia/macrophages both in resting (no-LPS) and activated (LPS) conditions (Figure 2). As time increased, the population of microglia/macrophages internalizing dendrimers increased from ~15% to ~80% within 3 h (Figure 2, Figure 3 and Figure 4A). Dendrimer uptake by microglia/macrophages peaked at 6 h after incubation with ~95% of the population positive for D-Cy5 signal both in flow cytometry (Figure 4A) and confocal microscopy (Figure 5). The dendrimers were retained in microglia/macrophages up to 24 h post-dendrimer incubation. In contrast, no dendrimers were found co-localized or internalized by any GFAP-positive astrocytes or other cell populations in mixed glial culture until 6 h post-dendrimer incubation. We found that ~8.5% of the total astrocyte population internalized dendrimers after 24 h of dendrimer (D-Cy5) incubation (Figure 4C).

### 3.2. Differential Uptake of Dendrimers by Microglial Cells

To determine if there are any differences in the rate and amount of dendrimer uptake between resting and activated microglial cells, we treated the cells with LPS (100 ng/mL) for 6 h to ‘activate’ the microglia/macrophages to a pro-inflammatory phenotype (to ‘mimic’ neuroinflammation in vivo). At 30 min post-dendrimer treatment, both the ‘resting’ and LPS-activated microglia/macrophages population showed similar dendrimer uptake (~15% and ~12% of cells, respectively) (Figure 4A and Figure 5). We also used the mean fluorescence intensity (MFI) to measure the amount of fluorescence in the cells. The MFI is proportional to amount of dendrimer (D-Cy5) in the cells (Figure 4B). At thirty minutes post-dendrimer incubation, the MFI was also similar for both activated and resting microglia/macrophages. One-hour post-dendrimer incubation, a greater percentage of the activated microglia/macrophages population (~38%) demonstrated dendrimer uptake, compared to non-LPS treated cells (~28%) (F(4,30) = 292.2, *p* < 0.01, *n* = 4) (Figure 4A).

**Figure 6:**
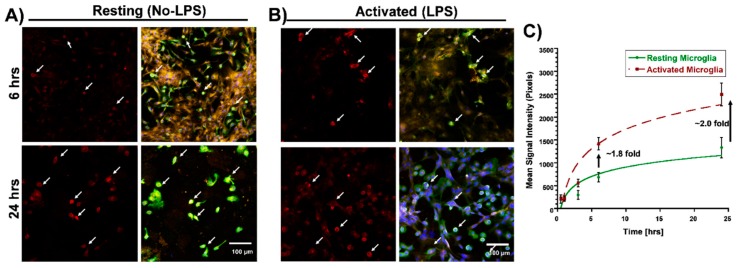
**Time-dependent (6 and 24 h) and differential dendrimer uptake between resting and activated microglial cells in mixed glial cultures from neonatal rabbit brain.** (**A**) Resting (no-LPS) microglia show time-dependent increase in dendrimer uptake (white arrows). (**B**) Activated (LPS) microglial cells show higher uptake of D-Cy5 (white arrows) compared to resting microglial cells, and this phenomenon is consistent as the time increases. (**C**) Mean signal intensity measurements of intracellular dendrimers (D-Cy5) form confocal images, suggesting relatively higher magnitude of dendrimer (D-Cy5) uptake in activated microglial cells (n = 4). Scale bar 100 µm.

MFI measurements also confirmed higher amounts of intracellular dendrimer uptake (~1.8-fold increase) compared to non-LPS treated cells (Figure 4B). Interestingly, more than 80% of the LPS-activated microglia/macrophage population demonstrated dendrimer uptake at 3 h after dendrimer treatment (Figure 4A). In contrast, ‘resting’ microglial cells demonstrated dendrimer uptake in only ~58% of the total microglial cell population (F(4,30) = 9.22, *p* < 0.01, n = 4) (Figure 4A and Figure 5). At 24 h, the proportion of microglia/macrophages population demonstrating dendrimer uptake was almost equivalent in both activated and ‘resting’ cells (Figure 4A and Figure 6). MFI measurements suggest that LPS-treated microglia have increased intracellular fluorescence (in other terms, greater intracellular dendrimer amount) at 1, 3, and 6 h post D-Cy5 treatment compared to non-LPS treated cells (F(4,30) = 2.11, *p* < 0.01, n = 4) (Figure 4B). Qualitative measurements of the intracellular fluorescence from the confocal images demonstrate that there is ~2-fold increase in magnitude of D-Cy5 uptake in LPS activated microglial cells (Figure 5 and Figure 6).

Astrocytes showed a delayed and limited uptake at 24 h, which was observed both in LPS and non-LPS treated groups. We did not observe dendrimer uptake in GFAP-positive cells (astrocytes), irrespective of LPS treatment until 6 h post-dendrimer incubation. At 24 h, ~8% of the total astrocyte population exhibited dendrimer uptake in both LPS and non-LPS treated cells (Figure 4C).

### 3.3. Effect of Inhibitors on Dendrimer Uptake in Primary Mixed Glial Cells

**Figure 7:**
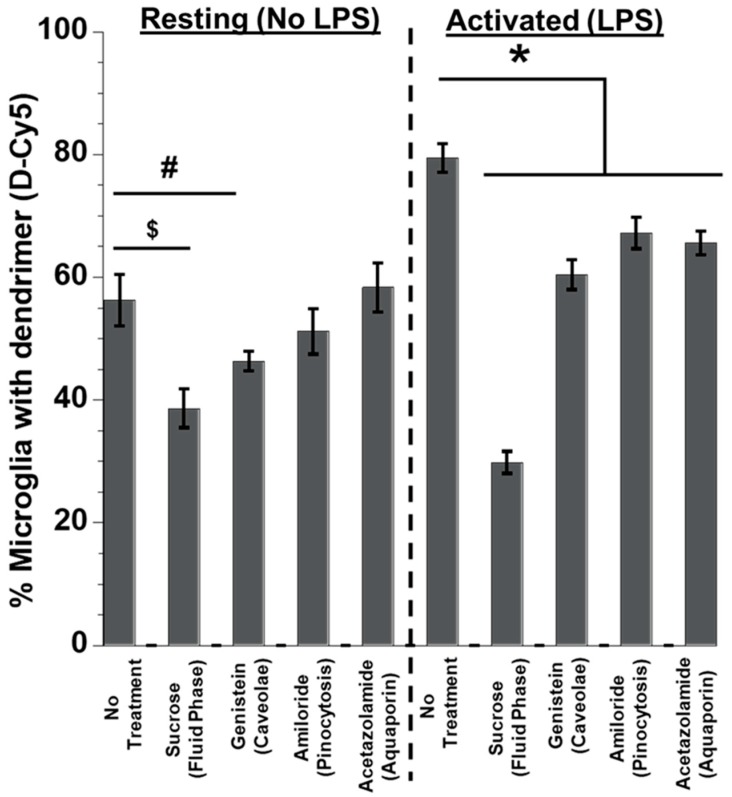
**Evaluation of dendrimer uptake using flow cytometry after treatment with various cell entry pathway inhibitors.** The mixed glial cultures were activated with LPS, various cell entry pathways were blocked using inhibitors, and the D-Cy5 uptake changes were evaluated using flow cytometry. D-Cy5 uptake was significantly reduced after blocking different endocytosis pathways (fluid phase and Caveolae-mediated endocytosis), suggesting that the cell entry of dendrimer majorly happens via endocytosis. In the case of LPS-activated cell population, D-Cy5 uptake was significantly inhibited when pinocytosis, aquaporin, and mannose pathways were blocked, suggesting that dendrimers may employ multiple pathways for cell entry in activated microglia. All the values are represented as mean ± SEM, n = 4, ^$^ *t*-test, *p* <0 .01, and ^#^ *t*-test *p* < 0.001. * Two-way ANOVA, *p* < 0.001 (no-LPS or LPS treated (Resting or Activated) no inhibitors (no treatment) (vs.) no-LPS or LPS-treated (resting or activated) with inhibitors).

We next investigated the mechanisms underlying the entry of dendrimers into microglia based on the microglial activation state. Previously, our group investigated the cell uptake mechanism of PAMAM dendrimers with different surface groups (Neutral -OH, Cationic -NH_2_, and anionic -COOH) by human lung adenocarcinoma epithelial cells (A549) cells [21]. Neutral dendrimers’ cell entry was shown to be primarily facilitated by non-caveolae, non-clathrein fluid phase endocytosis. In this current study, we investigated the uptake mechanism by the microglial population in rabbit brain primary mixed glial cells to see the effect of activation on dendrimer uptake. We treated the cells with various inhibitors to block the cell entry pathways. Cell viability above 85% was considered to be acceptable (Supplementary Figure S1). None of the treatments decreased primary mixed glial cell viability beyond 85%. Since the size of dendrimers ranges from 4–10 nm (G3-G10), we hypothesized that dendrimers were taken up by the cells via endocytosis and pinocytosis. We used sucrose (450 nM) to inhibit fluid phase endocytosis. Sucrose treatment lowered dendrimer uptake in both resting (~40% lower) and, to a higher extent, in activated microglia (~60% lower) (F(6,42) = 2.66, *p* < 0.01, n = 4) (Figure 7). The magnitude of decrease in percent of microglia was greater upon sucrose treatment in the activated cells (~2.5-fold decrease vs ~1.6-fold decrease in resting cells (F(6,42) = 2.35, *p* < 0.01, n = 4) compared to the no treatment group (No LPS and LPS only). MFI measurements also show that the amount of dendrimer taken up by the cells was significantly decreased (~3-fold decrease in activated cells vs ~2-fold decrease in resting cells) compared to the resting microglial cells (*p* < 0.01) (Figure 7).

Upon blocking caveolae-mediated endocytosis using genistein, the percentage of microglia with D-Cy5 was decreased by ~17% in the ’resting’ cells. In activated microglia, inhibiting caveolae-mediated endocytosis resulted in a decrease of the microglia population containing D-Cy5 by ~25% (Figure 7). Blocking pinocytosis, and aquaporin pathways did not significantly decrease the dendrimer uptake by ‘resting’ microglia, but there was a significant decrease in percent of activated microglia expressing dendrimer signal by ~15% (Amiloride, pinocytosis) (*p* < 0.05) and ~18% (Acetazolamide, aquaporins) (*p* < 0.05) compared to the no-treatment group, respectively (Figure 7). This suggests that activated microglia may be able to employ the above-mentioned pathways other than endocytosis for dendrimer uptake.

## 4. Discussions

Dendrimer-enabled drug delivery to activated glial cells is opening new opportunities for therapies in CNS disorders [4]. Understanding the mode by which dendrimers get internalized by glial cells will help in designing appropriate dendrimer-based drug delivery nanodevices and optimizing their use for multiple CNS applications, particularly in diseases in which neuroinflammation plays a role. Many studies have shown that cationic (amine terminated) PAMAM dendrimers demonstrate cell toxicity and platelet aggregation, and partial masking of the surface amine groups reduces the neurotoxicity [31,32]. In contrast, neutral dendrimers show low cell toxic profile, which does not induce compliment activation nor platelet aggregation [33]. In this study, we used neutral, hydroxyl-terminated, generation 4 PAMAM dendrimers, which showed low cytotoxicity on primary glial cells, which was supported by earlier findings on different cell lines [16,34,35]. Along with D4-OH dendrimers, all of the cell entry pathway inhibitors had no effect on cell viability. These findings were similar to multiple in vitro and in vivo studies [36,37,38]. Glial cells are crucial for maintaining the homeostatic balance of the CNS and exhibit a surveillance function [39]. Among microglia/macrophages and astrocytes, microglial/macrophages cells are the ones accountable for recognition of invaders and immunological stimulators [40,41,42]. This may explain the more rapid and greater uptake of D4-OH by microglia rather than by astrocytes and other glial cell species in the mixed glial cell cultures. The earliest D4-OH PAMAM dendrimers primary glial cell internalization at 30 min was comparable to data previously reported previously by our group in immortalized BV2 mouse microglial cells [16,29]. Similar to our previous studies, we find that by 3 h most of the microglial cells have taken up the dendrimer [16,21,27]. It has been previously reported that dendrimer uptake is an active process, as decrease in temperature resulted in decrease in dendrimer uptake by ~90% [21]. These studies used cell lines, and in these the initial uptake is more rapid. However, the mechanisms of uptake are similar to those published by our group for A549 lung epithelial cells in which fluid phase endocytosis played a major role in the uptake of the neutral, hydroxyl terminated dendrimer [21]. 

In this work, we further illustrated differences in the cellular uptake kinetics of dendrimers between resting and activated primary microglial cells. LPS-activated microglia/macrophages showed higher dendrimer uptake compared to non-LPS-treated cells. This can be attributed to increased endocytosis in activated microglia/macrophages [43,44]. LPS treatment activates the toll-like receptors (TLR) pathway, which has been reported previously to increase endocytosis in microglial cells [45,46,47], and which can contribute to a higher dendrimer uptake. Astrocytes, irrespective of their activation state, exhibited limited and delayed dendrimer uptake in primary mixed glial culture. We have previously studied the cellular uptake pattern of D4-OH dendrimers in vivo at 24 h after subarachnoid injections [26]. The results are in good agreement with our current study demonstrating delayed and relatively lower uptake in astrocytes [48]. Mechanisms of cell uptake in the astrocytes are not studied here and will be explored in the future. It is possible that other mechanisms including exosomal or microsomal transport may occur between microglia and astrocytes that transport the dendrimer to astrocytes [49].

Endocytosis is an active cell transport mechanism that cells primarily adopt to internalize extracellular materials through the plasma membrane [50,51]. In the current scenario, our results indicate that fluid phase endocytosis constitutes the main pathway for internalization for D4-OH PAMAM dendrimers in microglial cells. Several studies have been reported that activation of microglia via various stimulants such as LPS, IL-4, and INF-γ will enhance various cell uptake mechanisms such as fluid phase micropinocytosis, caveolae-mediated endocytosis, and phagocytosis [44,52,53,54]. Therefore, other independent endocytosis mechanisms such as caveolae, clathrein-mediated endocytosis, and macropinocytosis, which can also play a significant role in cellular internalization, should be taken into account [55,56]. Glucose treatment resulted in significant inhibition of dendrimer uptake in both resting and activated microglia/macrophages. These data are similar to our previous studies in a lung epithelial cell line (A549) in which non-specific fluid phase endocytosis (hypertonic sucrose inhibition) had a major role to play in cell uptake of the neutral, hydroxyl-terminated D4 PAMAM dendrimer. On the other hand, it is important to note that exposing the cells to hypertonic conditions may result in non-selective inhibition of other cell uptake pathways, particularly in activated cells. Blocking caveolae-mediated endocytosis using genistein also impeded dendrimer uptake in activated microglia. This can be attributed to an increase in endocytosis activity in activated microglial cells; therefore, blocking endocytosis pathways resulted in decreased dendrimer uptake. The aforementioned pathways have been well documented as modes of cellular entry for hydroxyl-terminated dendrimers in different cell lines [21,28,36,51]. Some of the most integral membrane proteins, which bring extracellular water and small solutes to the internal compartments of cells, are the aquaporin channels [57]. Blocking aquaporin channels using acetazolamide resulted in reduced dendrimer uptake in LPS-activated microglial cells. It has to be taken into account that LPS is known to downregulate certain types of aquaporin channels [58]. On the other hand, we cannot exclude other receptor pathway such as mannose receptors, toll-like receptors (TLR), macrophage scavenger receptors (MSR-1), or other complement receptors that are also reported to be upregulated in activated microglia/macrophages [59]. Additional studies investigating transport in specific knockout animals will help to fully characterize potential receptor candidates on the microglia/macrophage surface. Our results suggest that microglia/macrophages in their activated state may use more than one pathway that potentially contributes to dendrimer targeting and its cellular internalization. Since microglia/macrophages are implicated in multiple CNS neuroinflammatory diseases such as multiple sclerosis, cerebral palsy, Alzheimer disease, Parkinson’s disease, and amyotrophic lateral sclerosis (ALS), appropriate design and manipulation of studied dendrimer nanodevices may be harnessed as potential treatment options for diverse CNS diseases.

## 5. Conclusions

Our study demonstrates early uptake of D4-OH dendrimers exclusively by primary microglia/macrophages in mixed glial cultures, compared to astrocytes. Activated microglia showed faster and increased dendrimer uptake when compared to resting microglia, although the overall extent of uptake at 24 h was similar between resting and activated microglia. At 24 h, astrocytes displayed restricted uptake at a similar rate in both the resting and activated cells. Both resting and activated microglial/macrophage cells primarily employ fluid phase endocytosis for dendrimer uptake. Activated microglial cells appear to employ more than one mechanism to take up the hydroxyl-terminated generation 4 dendrimer. This can be attributed to increased overall endocytotic processes in the pro-inflammatory microglia/macrophages. Cellular dendrimer uptake can also happen via aquaporin channels, but further studies and other modes of activation are necessary to clarify the role of aquaporin channels and other receptor pathways such as mannose in D4-OH PAMAM dendrimer uptake by primary glial cells. These findings may give us important insights for improving the selective targeting characteristics of dendrimers and delivering therapeutic agents to microglia for attenuating neuroinflammation in a wide range of CNS diseases.

## Supplementary Materials

The following are available online.
