# Supplementary material for: Preferential and Increased Uptake of Hydroxyl-Terminated PAMAM Dendrimers by Activated Microglia in Rabbit Brain Mixed Glial Culture
Source: Molecules. 2018 Apr 27;23(5):1025. doi: 10.3390/molecules23051025 (PMC6102539; doi:10.3390/molecules23051025)
Supplement: Supplementary file 1 [file molecules-23-01025-s001.pdf]

Supplementary Section

Preferential and increased uptake of hydroxyl terminated PAMAM dendrimers by activated microglia in rabbit brain mixed glial culture

Yossef Alnasser † [1,4], Siva P. Kambhampati † [2], Elizabeth Nance [1,5], Labchan Rajbhandari [3], Shiva Shrestha [3], Arun Venkatesan [3], Rangaramanujam M Kannan* [2], Sujatha Kannan*[,1,2].

[1]Department of Anesthesiology and Critical Care Medicine, Johns Hopkins University School of Medicine, Baltimore, MD

[2]Center for Nanomedicine, Wilmer Eye Institute, Department of Ophthalmology, Johns Hopkins, University School of Medicine, Baltimore, MD

[3]Department of Neurology, Johns Hopkins University School of Medicine, Baltimore, MD, USA

[4]Current Address: Department of Pediatrics, BC Children's Hospital, University of British Columbia, Vancouver, BC, Canada.

[5]Current Address: Department of Chemical Engineering, University of Washington, Seattle, WA, USA.

† Co-first authors with equal contribution to this work

*****Corresponding authors:

Sujatha Kannan, Department of Anesthesiology and Critical Care Medicine, Johns Hopkins University, Baltimore, MD-21205, USA.

Tel.: +1 410 955 7610; email: skannan3@jhmi.edu

Rangaramanujam M. Kannan, Department of Ophthalmology, Center for Nanomedicine at the Wilmer Eye Institute, 400 North Broadway, Baltimore, Maryland 21231, USA

Tel.: +1 443-287-8634; e-mail: krangar1@jhmi.edu.

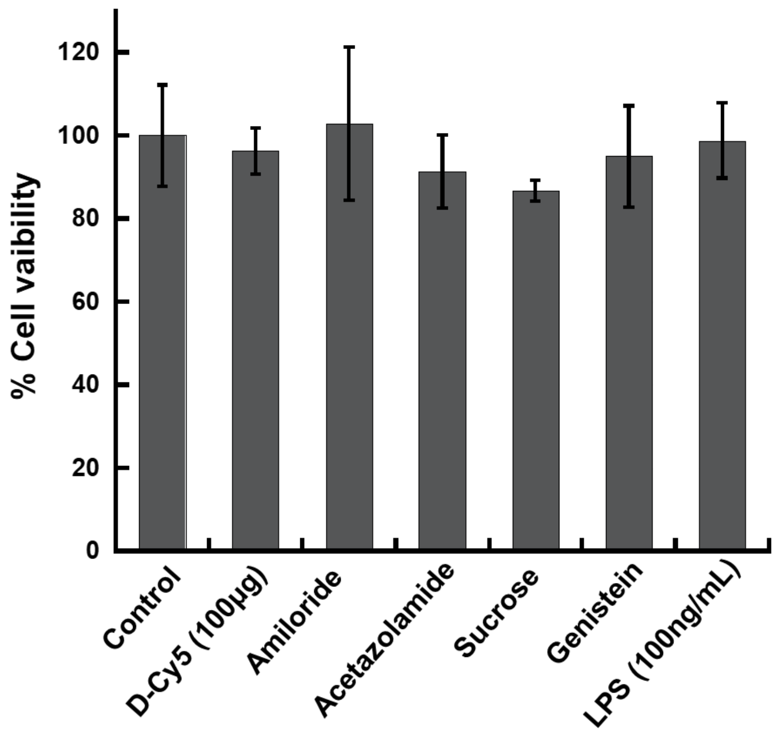

Figure S1: Cell toxicity profile various inhibitors in primary mixed glia culture. The various treatments D-Cy5 (100 µg/mL), Amiloride (10µM) to prevent macropinocytosis, Acetazolamide (100 nM) obstruct aquaporin channels, Sucrose (450 nM) to impede fluid phase endocytosis, Genistein (100 nM) to block caveolae-mediated endocytosis, and LPS (100 ng/mL) to activate the cells were evaluated for cytotoxicity. Three hours treatment with aforementioned treatments did not demonstrate significant cytotoxicity (>85%).
